# Utility of diffusion tensor imaging for guiding the treatment of lumbar disc herniation by percutaneous transforaminal endoscopic discectomy

**DOI:** 10.1038/s41598-019-55064-3

**Published:** 2019-12-10

**Authors:** Jiaqi Li, Hao Cui, Zhipeng Liu, Yapeng Sun, Fei Zhang, Yingcai Sun, Wei Zhang

**Affiliations:** 1 Department of Spinal Surgery, The Third Hospital of Hebei Medical University, Hebei, China; 2 Department of Medical Image, The Third Hospital of Hebei Medical University, Hebei, China

**Keywords:** Spinal cord diseases, Outcomes research

## Abstract

The purpose of this study was to evaluate the utility of diffusion tensor imaging (DTI) for guiding the treatment of lumbar disc herniation (LDH) by percutaneous transforaminal endoscopic discectomy (PTED). We collected the clinical data of a total of 19 patients: 10 with unilateral S1 nerve root injury, 6 with unilateral L5 nerve root injury, and 3 with unilateral L5 and S1 nerve root injury. All patients underwent DTI before surgery, 3 days post-surgery, 30 days post-surgery, and 90 days post-surgery. The comparison of the fractional anisotropy (FA) values of compressed lateral nerve roots before surgery and 3, 30, and 90 days post-surgery demonstrated the recovery of nerve roots to be a dynamic process. A significant difference was found in the FA values between compressed lateral nerve roots preoperatively and normal lateral nerve roots before surgery, 3 days post-surgery and 30 days post-surgery (*p* < 0.05). There was no significant difference in FA values between compressed lateral nerve roots and normal ones 90 days post-surgery (*p* > 0.05). DTI can be used for the accurate diagnosis of LDH, as well as for postoperative evaluation and prognosis, and it is thus useful for the selection of surgical timing.

## Introduction

In recent years, percutaneous transforaminal endoscopic discectomy (PTED) has been widely used in the treatment of lumbar disc herniation (LDH), with very good clinical effects^1^. Patients are increasingly demanding minimally invasive treatment for LDH. Moreover, with the general aging of society, elderly patients with multiple systemic diseases and in poor physical condition who cannot tolerate open surgery have become highly frequent; therefore, precision has become increasingly important in minimally invasive treatments. In this context, clinicians mainly rely on tools such as the Visual analogue scale (VAS), Japanese orthopaedic association scores (JOA), and Oswestry disability index (ODI), along with other indicators to evaluate surgical effects. Nevertheless, these indicators are greatly affected by patients’ subjective feelings, and cannot objectively evaluate surgical effect and expected recovery.

Magnetic resonance imaging (MRI) is widely used to diagnose LDH, as it provides valuable information, such as the position and size of the herniation. However, in clinical practice, the degree of nerve root compression observed in MRI is often inconsistent with the patient’s symptoms. Therefore, MRI is unable to accurately identify the cause of symptoms and quantitatively evaluate nerve injury^2^. Magnetic resonance diffusion tensor imaging (DTI) is a non-invasive method for tracing nerve fiber tracts and evaluating nerve injury. It is also a method for indirectly observing the change process of nerve tissue^3–6^. DTI can be used to display peripheral nerve and the decrease of average FA value indicates the existence of demyelination injury^6^. A list of ‘Do’s and Don’ts’ was made by Jones *et al*.^7^ to make DTI better reflecting the actual neurological change. Thus, DTI can more accurately diagnose LDH, clarify the affected segment and the degree of nerve injury, and monitor postoperative nerve root recovery. The purpose of this study was to evaluate the utility of DTI for guiding the surgical treatment of LDH.

## Materials and Methods

### Ethical statement

This study was approved by the ethics committee of The Third Hospital of Hebei Medical University (2017-019-1). All patients signed informed consent and allowed use of their clinical data for this study. All methods were performed in accordance with the relevant guidelines and regulations.

### Inclusion and exclusion criteria

The study subjects were patients with LDH who were treated with PTED in our hospital from June 2017 to April 2018. Patients initially included in this study met the following criteria: Patients with LDH (1) for whom conservative treatment had been ineffective and had typical symptoms of nerve root compression; (2) with protrusions confined to the L4-S1 segments; and (3) with no history of lumbosacral surgery of contraindications for MRI. Patients with bilateral symptoms were excluded from the study. All patients underwent DTI of lumbosacral nerve roots before surgery, as well as 3, 30 and 90 days post-surgery.

### Collection of data from nuclear magnetic resonance imaging

This study used a German Siemens Verio 3.0 T superconducting MR instrument and an 8-channel Spine Matrix coil. The subjects were in supine position, and the subject’s head and upper body were in a magnetic resonance coil. The location center was set 3–5 cm below the umbilicus. Plain MRI scanning was performed for sagittal plane positioning. Sagittal plane T2WI settings: TR = 3000 ms, TE = 101 ms, scanning slice number = 11, slice thickness = 4 mm, FOV = 300.0 mm × 300.0 mm, voxel = 1.0 mm × 0.8 mm × 4.0 mm, and number of excitations = 2. Horizontal axis T2WI settings: TR = 3500 ms, TE = 112 ms, scanning slice number = 10, slice thickness = 3 mm, FOV = 220.0 mm × 220.0 mm, voxel = 0.9 mm × 0.7 mm × 3.0 mm, and number of excitations = 1. The settings for the 3D short-time inversion recovery fast spin echo (3D STIR SPACE) coronal scan were: TR = 3800 ms, TE = 309 ms, slice thickness = 1 mm, FOV = 250.0 mm × 250.0 mm, voxel = 0.8 mm × 0.8 mm × 1.0 mm, echo chain length = 167, and number of excitations = 1.5; no flow compensation. The scanning range covered from the upper margin of L2 to the lower margin of S3. The settings for the DTI cross-sectional scanning were: TR = 8000 ms, TE = 95 ms, slice thickness = 4 mm, scanning slice number = 40, FOV = 350.0 mm × 350.0 mm, voxel = 2.7 mm × 2.7 mm × 4.0 mm, diffusion direction = 12, b value = 0.800 s/mm^2^, bandwidth = 1396 Hz, echo spacing = 0.78 ms, and number of excitations = 5; the scanning range covered from the upper margin of L4 to the level of S3. We uploaded 3D STIR SPACE image and DTI tensor image to Siemens Verio Neuro 3D workstation to fuse the two. We used 3D STIR SPACE image to provide anatomical information for FA value measurement. The nerve root was divided into proximal, middle and distal segments by the entrance and exit of the intervertebral foramen. L5 nerve roots were measured and region of interests (ROIs) were performed at the three positions of “posterior superior margin of L5 vertebra”, “outlet of intervertebral foramen” and “anterior inferior margin of L5 vertebra”. The nerve roots of S1 were measured and ROIs were performed at the three positions of “posterior superior margin of S1 vertebra”, “posterior inferior margin of S1 vertebra” and “anterior inferior margin of S1 vertebra”. DTI parameters of nerve roots in ROIs were obtained. The FA threshold was set to 0.18, the maximum inversion angle was 30, and each ROI of L5-S1 nerve root accounted for 2–4 voxels. It ensured that each voxel had nerve passage, avoiding errors caused by manual depiction of ROI, and reduced the impact of partial volume effect, thus making the measured ROI data more reliable. The mean FA value of ROIs in the proximal, middle and distant position of each nerve was taken as the final FA value of the corresponding nerve. FA values of affected and contralateral nerve roots were recorded in each patient before surgery, and 3, 30, and 90 days post-surgery, respectively. The FA values were measured by two participants and the final result is averaged between the two. If there was a large difference between values measured by the two participants, the third participant measured again and the final result was averaged between correct two.

### Data analysis

SPSS v17.0 was used for statistical analysis. The normally distributed measurement data was expressed with mean ± standard deviation. Otherwise, they were represented with median (interquartile range). The paired sample t-test was used to compare the FA values of damaged nerve roots and contralateral undamaged nerve roots of patients in the two groups before surgery, and 3, 30, and 90 days post-surgery, respectively. ANOVA was used to compare dynamic changes in the FA values of the damaged nerve roots before surgery, and 3, 30, and 90 days post-surgery. We performed correlation analysis between duration of symptoms and FA values. Results with *p* < 0.05 were considered statistically significant.

## Results

Demographic data for the patients was showed in Table 1. A total of 19 patients, 12 males and 7 females, with an average age of 35 years (26–53 years) were enrolled in this study. According to the preoperative localization of the damaged nerve roots, the 19 patients were divided into a group with damaged S1 nerve roots (S1 group, n = 13), and a group with damaged L5 nerve roots (L5 group, n = 9). In 3 patients, ipsilateral L5 and S1 nerve root damage was found simultaneously, yet the preoperative MRI showed single-segment LDH. Therefore, these 3 patients were classified as L5 group or S1 group with L5 nerve root damage or S1 nerve root damage, respectively.

**Table 1:** Basic information of patients.

Values	
Age (year)	35 (16)
Sex (male/female)	12/7
Duration of symptoms (month)	3 (10)
**Damaged nerve roots**	
L5	6 (31.6%)
S1	10 (52.6%)
L5 and S1	3 (15.8%)

**Figure 1:**
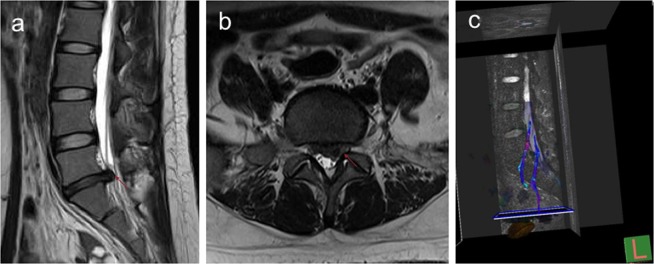
Preoperative lumbar disc MRI and lumbosacral nerve root DTI scan. (**a**) is the sagittal position of the patient’s preoperative MRI. (**b**) is the axial position of the patient’s preoperative MRI, and the protruding intervertebral disc is the left area II. (**c**) is the nerve root reconstructed by DTI before surgery, indicating obvious changes in the microstructure of the compressed nerve root.

Comparison of FA values between the affected side and uninjured side nerve roots were shown in Table 2. The preoperative mean FA values of the affected side and uninjured side nerve roots in the S1 group were 0.359 ± 0.023 and 0.460 ± 0.029, respectively. On the other hand, the preoperative mean FA values of the affected side and uninjured side nerve roots in the L5 group were 0.354 ± 0.031 and 0.448 ± 0.037, respectively. The preoperative FA values of the affected side were significantly lower than those of the uninjured side (*p* < 0.05). Preoperative lumbar disc MRI and lumbosacral nerve root DTI scan was showed in Fig. 1.

**Table 2:** Comparison of FA values between the affected side and uninjured side nerve roots.

FA Values		Pre-op	3 days post-op	30 days post-op	90 days post-op	*P* (2-tailed)
FA (L5)	affected side	0.354 ± 0.031_a_	0.379 ± 0.029_ab_	0.408 ± 0.035_bc_	0.421 ± 0.018_c_	<0.001^#^
	uninjured side	0.448 ± 0.037	0.479 ± 0.042	0.462 ± 0.036	0.442 ± 0.034	—
	*P* (2-tailed)	<0.001^$^	0.001^$^	0.008^$^	0.38^$^	—
FA (SI)	affected side	0.359 ± 0.023_a_	0.367 ± 0.033_a_	0.383 ± 0.031_a_	0.437 ± 0.024_b_	<0.001^#^
	uninjured side	0.460 ± 0.029	0.454 ± 0.039	0.458 ± 0.040	0.445 ± 0.025	—
	*P* (2-tailed)	<0.001^$^	<0.001^$^	<0.001^$^	0.134^$^	—

FA indicated fractional anisotropy. ^#^, repeated measurement analysis of variance. ^$^, paired t-test. Multiple comparisons of each variable at different time points were used bonferroni method, and at least one identical subscript letter (a, b and c) denoted no significant difference from each other.

**Figure 2:**
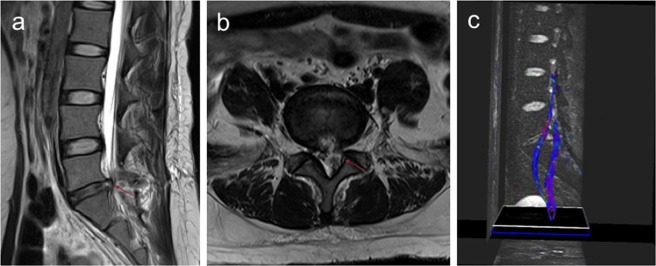
Lumbar disc MRI and lumbosacral nerve root DTI scan at 3 days post-surgery. (**a**) is the sagittal position of the patients’ MRI 3 days post-surgery, and the herniation has disappeared, leaving the posterior longitudinal ligament with the “grape skin” phenomenon. (**b**) is the axial position of the patient’s MRI 3 days post-surgery, adequate spinal canal decompression can be observed. (**c**) is the nerve root reconstructed by DTI 3 days post-surgery, no obvious recovery of the compressed nerve microstructure can be observed.

The mean FA values of the affected side and the uninjured side nerve roots 3 days post-surgery in the S1 group were 0.367 ± 0.033 and 0.454 ± 0.039, respectively. In contrast, the mean FA values of the affected side and the uninjured side nerve roots 3 days post-surgery in the L5 group were 0.379 ± 0.029 and 0.479 ± 0.042, respectively. The mean FA values of the affected side were significantly lower than those of the uninjured side 3 days post-surgery (*p* < 0.05). Lumbar disc MRI and lumbosacral nerve root DTI scan was showed in Fig. 2.

**Figure 3:**
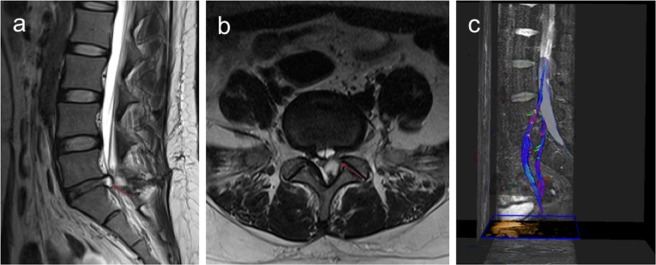
Lumbar disc MRI and lumbosacral nerve root DTI scan at 30 days post-surgery. (**a**) is the sagittal position of the patient’s MRI 30 days post-surgery, showing an obvious “grape skin” phenomenon. (**b**) is the patient’s MRI axial position 30 days post-surgery, showing the spinal canal is not compressed. (**c**) is the nerve root reconstructed by DTI 30 days post-surgery, partial recovery of the compressed nerve root microstructure was observed.

Furthermore, the mean FA values of the affected side and the uninjured side nerve roots 30 days post-surgery in the S1 group were 0.383 ± 0.031 and 0.458 ± 0.040, respectively; whereas the mean FA values of the affected side and the uninjured side nerve roots 30 days post-surgery in the L5 group were 0.408 ± 0.035 and 0.462 ± 0.036, respectively. We found the mean FA values of the affected side were significantly lower than those of the uninjured side 30 days post-surgery (*p < *0.05). Lumbar disc MRI and lumbosacral nerve root DTI scan was showed in Fig. 3.

**Figure 4:**
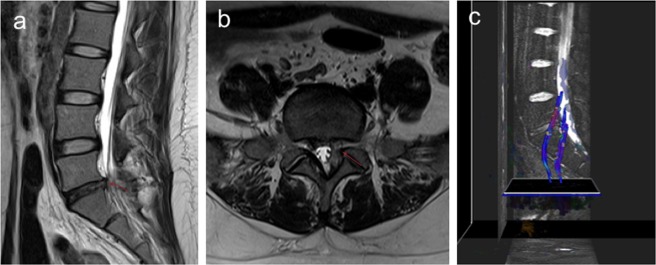
Lumbar disc MRI and lumbosacral nerve root DTI scan at 90 days post-surgery. (**a**) is the patient’s MRI sagittal position 90 days post-surgery, the posterior longitudinal ligament is reattached, and the “grape skin” phenomenon has basically disappeared. (**b**) is the patient’s MRI axial position 90 days post-surgery, showing the spinal canal is well dilated. (**c**) is the nerve root reconstructed by DTI 90 days post-surgery, and the compressed nerve root microstructure is fully restored, comparable to the uninjured side.

Finally, the mean FA values of the affected side and the uninjured side nerve roots 90 days post-surgery in the S1 group were 0.437 ± 0.024 and 0.445 ± 0.025 (*p* = 0.134), respectively; while the mean FA values of affected side and the uninjured side nerve roots 90 days post-surgery in the L5 group were 0.421 ± 0.018 and 0.442 ± 0.034 (*p* = 0.38), respectively. No statistically significant difference was found between the FA values of the affected and uninjured sides 90 days post-surgery. Lumbar disc MRI and lumbosacral nerve root DTI scan was showed in Fig. 4.

**Figure 5:**
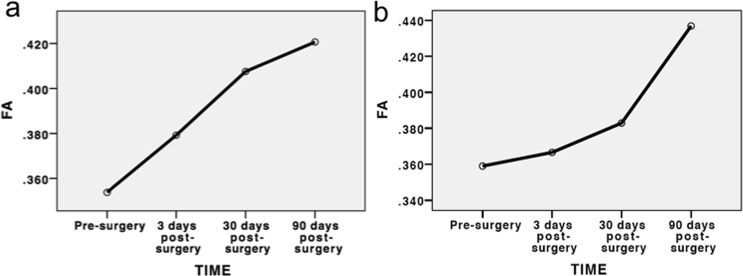
(**a**) Dynamic changes of mean FA values of the affected nerve root in L5 group. (**b**) Dynamic changes of mean FA values of the affected nerve root in S1 group.

The differences in FA values of affected side in the S1 group were significantly different at pre-surgery, and 3, 30, and 90 days post-surgery (Table 2). In particular, a statistically significant difference was found between the FA value at 90 days post-surgery and the values in the preoperative evaluation, and 3, 30 days post-surgery, respectively (all *p* < 0.001). On the other hand, the FA values of affected side in the L5 group between different time points were significantly different (p < 0.001). At 90 days post-surgery, the difference was statistically significant compared with the preoperative values and 3 days post-surgery, respectively (all p < 0.001). In summary, we demonstrated the recovery of nerve roots to be a gradual process (Fig. 5). Spearman correlation analysis indicated that FA values were not related to duration of symptoms (*p* = 0.208).

## Discussion

Diffusion tensor imaging is a novel technique developed after the diffusion weighted imaging (DWI). DTI can detect the random movement of water molecules in nerve tissue, as well as measure their anisotropy, quantitatively analyze the diffusion of water molecules in nerve tissue with FA values, and evaluate the change of peripheral nerves^3,8^. Furthermore, DTI is a method that can indirectly assess nerve tissue microstructure and noninvasively display nerve fiber bundle angiography^3–6^. Researches have shown that DTI can indirectly evaluate the degeneration and regeneration of peripheral nerves^9–11^. In addition, Oswestry disability index (ODI) scores and symptom duration have been negatively correlated with FA value and positively correlated with apparent diffusion coefficient (ADC) value of compressed nerve roots in patients with LDH^12^. correlation analysis in this study did not found significant correlation between duration of symptoms and FA values. This might be due to the small sample size of this study. This study focused on FA values, so no clinical symptoms evaluation was analyzed. The decrease in FA values in this context has been attributed to histological changes in the nerve root, including microstructure modifications such as demyelination of nerve fibers, loss of axons, neurofibrosis and degeneration, resulting in an increase of isotropic water volume^13–15^. The sensitivity and specificity of FA values for the detection of nerve injury have been reported to be higher than those of ADC values (73.3% and 100% vs. 13.4% and 80%). FA values appear to be superior to ADC values in the diagnosis of lumbosacral radiculopathy, with potential application for post-treatment evaluation^2,16–19^. Therefore, in this study, we used FA values for preoperative diagnosis, postoperative evaluation and prognosis of patients with LDH.

When DTI is used to distinguish between intravertebral canal stenosis and intervertebral foramen stenosis, changes in DTI indexes are related to the location of the nerve root compression. The decrease in FA value indicates the presence of intervertebral foramen stenosis and allows identification of the compressed nerve roots^20^. However, when patients’ symptoms are inconsistent with imaging findings, or when MRI reveals multi-stage disc herniation, accurate diagnosis can reduce the degree of decompression, limit surgical trauma, and diminish the cost of hospitalization, while also ensuring the surgical effect. Recent studies have shown the application of MRI combined with DTI or paraspinal mapping (PM) can reduce false positives on MRI, and also replace invasive methods such as discography and selective nerve root block to further enhance diagnosis^19^. In this study, we applied DTI to improve diagnosis instead of selective nerve root closure, with good results. Preoperative routine MRI revealed the presence of single-segment LDH in 3 patients, yet FA values showed these patients had severe unilateral and bilateral nerve root damage. All 3 of these patients underwent PTED. After the procedure, symptoms disappeared, the DTI index of both nerve roots significantly improved, and no significant differences were observed between these and those on the uninjured side. Accurate diagnosis of LDH combined with minimally invasive treatment avoids extensive decompression in conventional open surgery, and reduces the complexity and risk of surgical procedures. Studies have shown FA values and patients’ symptoms do not appear to improve significantly after conservative treatment^17^. Likewise, a long duration of preoperative symptoms has been reported to be an important factor affecting patients’ quality of life and prognosis, and early surgical intervention is recommended^21–24^. Therefore, we believe DTI can be used for the accurate diagnosis of LDH. Thus, if conservative treatment fails and DTI examination indicates a significant decrease in FA value, early surgical treatment is recommended.

Diffusion tensor imaging is a noninvasive method for effectively tracing nerve fiber bundles, as well as quantitatively evaluating nerve injury and axon regeneration^11,13,25^. Indeed, DTI is widely used in the clinical evaluation of nerve root lesions. Although research has shown a significant negative correlation between the FA value of compressed nerve roots and ODI scores^2^, few studies have evaluated the relationship between the dynamic changes of postoperative DTI indicators and the improvement of clinical symptoms. Therefore, all the patients in this study underwent PTED, and DTI scanning was performed before surgery, as well as 3 days, 1 month, and 3 months post-surgery, respectively. At the time of DTI reexamination 3 days post-surgery in both groups, the protruding nucleus pulposus completely disappeared in the sagittal and axial MRI images. However, the preoperative DTT reconstruction of the compressed nerve roots showed significant microstructural changes, with no significant improvement in these changes or FA values in the DTT reconstruction 3 days post-surgery. This may be due to the fact that although the procedure relieves the nerve root compression, the edema response caused by intraoperative stimulation of the nerve root remains. We believe this phenomenon explains why patients’ symptoms do not disappear immediately after the procedure. Comparison of the FA values before surgery, and 3, 30, and 90 days post-surgery revealed the differences between the two groups were statistically significant (*p* < 0.001). Indeed, we found the postoperative recovery of the compressed nerve roots was a dynamic process of gradual recovery. Patient A experienced slight symptom relief post-surgery, mild symptoms at the 1-month reexamination, and complete improvement at the 3-month reexamination. The changes in the DTI indexes were consistent with the evolution of the symptoms. Patient B had mild residual symptoms at the 3-month reexamination post-surgery, which was consistent with the incomplete improvement seen in the DTI index. We suggested open surgery for patient B, but they declined open surgery and chose to continue to a follow-up observation. When PTED is applied in cases with complicated protrusion of intervertebral discs, sufficient decompression of nerve roots cannot be accomplished. Residual nucleus pulposus and intervertebral disc tissue may continue to compress or stimulate nerve roots, leading to incomplete symptom relief. The non-significant improvement of FA values in patients post-surgery reflects the persistence of nerve root injury, indicating a poor prognosis. Therefore, we suggest open surgery should be performed as soon as possible in cases where PTED fails in the treatment of LDH.

This study has limitations. First, the relatively small sample size, meaning a large number of clinical studies is required to confirm repeatability of our results. Second, there was no control group. Third, the shift point position identity of the DTI values was poor.

In conclusion, we found DTI can be used for the accurate diagnosis, postoperative evaluation and progonosis of LDH, and is useful for guiding the selection of surgical timing.
